# Complex Regional Pain Syndrome

**DOI:** 10.31138/mjr.32.2.174

**Published:** 2021-06-30

**Authors:** Debashish Mishra, Arghya Chattopadhyay, Anwin Joseph Kavanal, Rajender Kumar, Shefali K. Sharma

**Affiliations:** 1 Clinical Immunology and Rheumatology Unit, Post Graduate Institute of Medical Education and Research, Chandigarh, India; 2 Department of Nuclear Medicine, Post Graduate Institute of Medical Education and Research, Chandigarh, India

**Keywords:** Complex regional pain syndrome, CRPS, reflex sympathetic dystrophy, causalgia, triphasic bone scan

**Figure 1:**
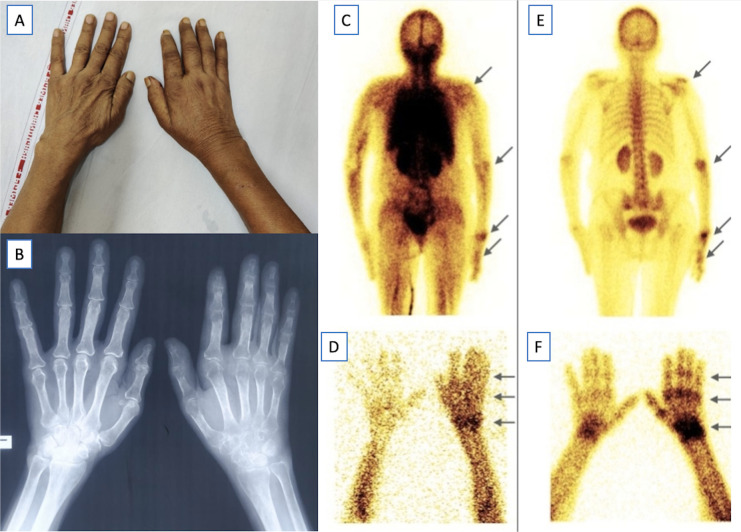
Diffuse swelling over right hand and forearm along with sparse hair (**A**), X-ray bilateral hands showing patchy osteopenia around right wrist and small joints of hand (**B**), ^99m^Tc-methylenediphosphonate (MDP) three phase bone scan: Blood pool phase images of whole body posterior view (**C**) and regional hand static dorsal view (**D**) showing relatively increased pooling of the tracer in the small joints of the right hand, right wrist, right elbow and right shoulder joint regions (arrows) compared to the left side; Delayed phase images of whole body posterior view (**E**) and regional hand static dorsal view (**F**) showing relatively increased uptake of the tracer (osteoblastic activity) in the periarticular regions of small joints of the right hand, right wrist, right elbow and right shoulder joint (arrows) compared to the left side. Image findings are suggestive of complex regional pain syndrome (CRPS) involving the right upper limb.

A 63-year-old woman presented with history of severe diffuse pain (even on touch) and swelling over right forearm and hand for 6 weeks. On inquiry, she gives history of past herpetic lesions over same side 4 months back. On examination, there was diffuse swelling, tenderness and warmth over right hand and forearm, along with increased sweating and loss of hair (**Figure 1A**). Radiograph showed patchy osteopenia around right wrist and small joints of hand (**Figure 1B**). Triple phase skeletal scintigraphy showed increased flow, perfusion and osteoblastic activity in right shoulder, elbow, wrist, and small joints of hand suggestive of complex regional pain syndrome (CRPS) (**Figure 1 C–F**). Her inflammatory markers (erythrocyte sedimentation rate and C-reactive protein) were normal, and rheumatoid factor, anti-nuclear antibody, and viral serologies (Human immunodeficiency virus, hepatitis B and C) were negative. Based on clinical and imaging findings, a diagnosis of CRPS triggered by herpes zoster infection was made. She was started on oral bisphosphonates along with non-steroidal anti-inflammatory drugs. On 4 weeks follow-up, she had marked improvement in pain and swelling.

Complex regional pain syndrome (CRPS) can be type I (without preceding nerve injury) or type II (with preceding nerve injury). It is characterised by diffuse severe burning pain with difficulty in movements, increased sweating, and vasomotor features like warmth and redness. Radiography and bone scan can aid in diagnosis, depending on the stage of disease. Treatment consists of analgesics, steroids, bisphosphonates, antidepressants, neuropathic medications, sympathetic blockade, spinal cord stimulation and dorsal root ganglion stimulation. However, to prevent CRPS by early mobilisation and graded physiotherapy along with psychological support is more beneficial than treatment.^1,2,3^
